# Schistosomiasis Burden and Trend Analysis in Africa: Insights from the Global Burden of Disease Study 2021

**DOI:** 10.3390/tropicalmed10020042

**Published:** 2025-02-03

**Authors:** Dandan Peng, Yajing Zhu, Lu Liu, Jianfeng Zhang, Peng Huang, Shaowen Bai, Xinyao Wang, Kun Yang

**Affiliations:** 1 School of Public Health, Nanjing Medical University, Nanjing 211166, China; pengdandan@stu.njmu.edu.cn (D.P.); huangpeng@njmu.edu.cn (P.H.); baisw2022@stu.njmu.edu.cn (S.B.); 2 Key Laboratory of National Health Commission on Parasitic Disease Control and Prevention, Jiangsu Provincial Key Laboratory on Parasite and Vector Control Technology, Jiangsu Institute of Parasitic Diseases, Wuxi 214000, China; 6222805018@stu.jiangnan.edu.cn (Y.Z.); liulu@jipd.com (L.L.); zhangjianfeng@jipd.com (J.Z.); 3 Wuxi School of Medicine, Jiangnan University, Wuxi 214000, China

**Keywords:** Africa, global burden of disease, machine learning model, spatial autocorrelation analysis, schistosomiasis, trend analysis

## Abstract

Schistosomiasis remains a major public health concern in Africa, despite global efforts to eliminate the disease by 2030. This study estimates the burden, trends, and inequalities of schistosomiasis in Africa from 1990 to 2021, and projects future prevalence to inform the WHO’s elimination strategies. Data from the Global Burden of Disease Study (GBD 2021) were used to calculate annual average percentage change (AAPC) and annual percentage change (APC), with spatial global autocorrelation analysis performed to examine temporal and spatial trends. Five modeling algorithms were constructed to predict disease burden in Africa from 2022 to 2041. The age-standardized prevalences rate (ASPR) of schistosomiasis in Africa decreased from 18,495.51 per 100,000 in 1990 to 9,461.76 per 100,000 in 2021. The total number of cases, disability-adjusted life-years (DALYs), and mortality accounted for 84.25%, 87.92% and 87.28% of the global totals, respectively. ARIMA modeling predicts that by 2030, the ASPR will reach 3.99%. Despite progress, the burden remains significant, and intensified efforts are needed, particularly in high-burden regions like West Africa, to meet the WHO’s 2030 elimination targets.

## 1. Introduction

Schistosomiasis, as one of the world’s most neglected tropical diseases, caused by the trematodes (flukes) of the genus *Schistosoma*, is the second most dangerous parasitic tropical disease after malaria [1,2]. The majority of infections are caused by *Schistosoma haematobium*, *Schistosoma japonicum* and *Schistosoma mansoni*, leading to urogenital and intestinal schistosomiasis, respectively [3]. The transmission of schistosomiasis depends on the excretion of eggs in the feces or urine of infected individuals, as well as the presence of snails, which serve as intermediate hosts. The larvae in snails produce thousands of free-swimming cercariae that penetrate human skin upon water contact [4], causing acute infections or recurrent chronic infections, which manifest symptoms such as hematuria, fever, pain and hepatosplenomegaly. In severe cases, schistosomiasis may result in cancer [5]. Schistosomiasis impacts not only the health of infected individuals through systemic and organ-specific inflammation, but also imposes social and economic burdens on communities [6], particularly in sub-Saharan African countries where *S. mansoni* and *S. haematobium* are prevalent. In addition to the common symptoms of hepatosplenomegaly caused by *S. mansoni*, urogenital schistosomiasis due to *S. haematobium* has been found to correlate with increased susceptibility to HIV, HPV infections and infertility [7]. Furthermore, long-term schistosomiasis infections, especially in children, can lead to growth retardation, fatigue, cognitive impairments and increased risk of anemia, contributing to poor academic performance, which further exacerbates socioeconomic burdens [8].

Schistosomiasis is endemic in 78 countries/regions worldwide, with recorded infections in Africa, Asia, the Middle East, and South America [9]. Among these, 52 countries experience moderate to high transmission level [10]. The disease is a leading cause of morbidity and mortality in Africa, South America, the Caribbean, the Middle East, and Asia [2], affecting approximately 779 million people globally and resulting in about 280,000 deaths annually [3]. Africa accounts for 93% of the approximately 207 million schistosomiasis cases worldwide, with the highest prevalence in Nigeria, Tanzania, Ghana, Mozambique, and the Democratic Republic of the Congo, totaling up to 78 million cases [3,10].

The WHO has long prioritized the control and elimination of schistosomiasis and has developed several roadmaps that have achieved significant progress. In its latest “2021–2030 Roadmap for Neglected Tropical Diseases”, the global targets include eliminating schistosomiasis as a public health problem in all endemic countries and interrupting transmission (no human cases) in selected countries [11]. To accelerate the progress toward the elimination of schistosomiasis, the WHO has recommended expanding the target groups for preventive chemotherapy, establishing water, sanitation, and hygiene programs, setting age group targets and frequencies for preventive chemotherapy, implementing health education and behavior change interventions, and conducting snail control activities [12]. However, the demand for preventive chemotherapy and the distribution of schistosomiasis in different regions remain unclear. Therefore, understanding and analyzing the prevalence and burden of schistosomiasis in Africa will better guide Mass Drug Administration efforts and support the achievement of schistosomiasis elimination targets.

The GBD study was established in 1991 and aims to quantify the severity of all major diseases, risk factors, and intermediate clinical outcomes in a highly standardized manner, allowing for comparisons across different times, populations and health issues [13,14]. Over the past 30 years, the GBD study has continually adjusted its assessment methods, expanded the coverage of disease risk factors, and focused on the best estimates and forecasts of disease burden. In recent GBD cycles, a series of health-related Sustainable Development Goals (SDG) forecasts have been incorporated, meeting the needs of health decision-makers for updated and decision-relevant health information. The GBD study is currently regarded as the most reliable and comprehensive study on disease burden worldwide [15]. The GBD 2021 study (www.healthdata.org), released in May 2024, evaluates the incidence, mortality and DALYs for 371 types of diseases and injuries across 204 countries and regions for both males and females from 1990 to 2021. The GBD project team has reported detailed research methodologies [16]. This study analyzes the temporal and spatial trends in the burden of schistosomiasis in Africa based on the GBD 2021 study, and constructs models to predict future burdens, providing recommendations to accelerate the achievement of the WHO’s schistosomiasis elimination goals in the African region.

## 2. Methods

### 2.1. Data Sources

All data for this study are derived from the GBD 2021 study. The data on schistosomiasis include survey data, disease registries, hospital data, insurance claims, and literature reviews, compiled by the Institute for Health Metrics and Evaluation (IHME) at the University of Washington. These data include the number of cases, DALYs, mortality, ASPR, ASDR and age-standardized mortality rates (ASMR), categorized by gender with a 95% UI. In the GBD 2021 study, schistosomiasis was identified according to the International Classification of Diseases, 10th Revision (ICD-10) (coding range: B65–B65.9).

### 2.2. Data Extraction and Processing

Data related to schistosomiasis, including prevalence, mortality, and DALYs data, were extracted from the GBD 2021 study. ArcMap 10.7 software was used to create maps covering 54 African countries to visualize the ASPR, ASDR and ASMR for the year 2021. Heat maps displayed trends in these indicators from 1990 to 2021, analyzing differences across regions and countries. The ASPR, ASMR and ASDR were calculated using the following formula: 

Age Standardized Rate (Per 100,000 population) =$\;\frac{\sum_{i=1}^{A}ai\times wi}{\sum_{i=1}^{A}wi}\times 100,000$

In this formula, ai represents the prevalence, DALY rate, or mortality rate for the i age group, and wi represents the population of the same age group in the GBD standard population. 

### 2.3. Descriptive Statistical Analysis

Joinpoint software (version 5.2.0, website: https://surveillance.cancer.gov/joinpoint/ accessed on 16 May 2024) was used to calculate the AAPC and the APC for ASPR, ASDR and ASMR. A trend analysis was conducted, categorizing trends as increasing (AAPC > 0), decreasing (AAPC < 0), or stable (when the 95% CI includes 0) [17].

### 2.4. Spatial Autocorrelation Analysis

Spatial autocorrelation analysis was conducted using ArcMap 10.7 to assess the spatial clustering of schistosomiasis burden indicators across African countries. The clustering distribution module in spatial statistics tools was selected to calculate the Moran’s I coefficient and the corresponding *p*-value. The spatial autocorrelation was determined based on the value of the Moran’s I coefficient—the global Moran’s I ranges from −1 to 1, where −1 indicates complete negative correlation, 0 indicates random distribution, and 1 indicates complete positive correlation. If Moran’s I > 0, it indicates positive spatial autocorrelation; if Moran’s I < 0, it indicates negative spatial autocorrelation; if Moran’s I = 0, it indicates random distribution [18].

### 2.5. Machine Learning Model Construction

For time series forecasting, this study implemented five machine learning algorithms using the R software to predict ASPR, ASDR and ASMR for the period 2021–2031. First, the extracted schistosomiasis data were formatted for model input. Next, five models were selected for prediction, as follows: Autoregressive Integrated Moving Average (ARIMA), Prophet, Multivariate Adaptive Regression Splines (MARS), Distributed Lag Linear Model (DLM) and Bayesian Structural Time Series (BSTS). Each model was trained and validated using historical data. Finally, model performance was compared using metrics such as Mean Squared Error (MSE), Root Mean Squared Error (RMSE) and Mean Absolute Percentage Error (MAPE) to select the best model for future predictions.

All statistical analyses were conducted using R software (version 4.4.1), with the significance level set at a *p*-value < 0.05.

## 3. Results

### 3.1. Continental Level

**Figure 1:**
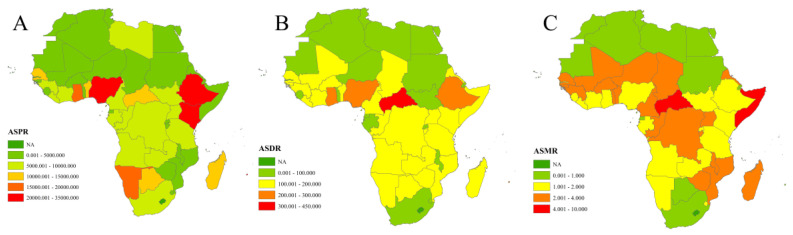
The age-standardized prevalences rate (**A**), age-standardized DALYs rate (**B**), and age-standardized mortality rate (**C**) of schistosomiasis for 54 African countries in 2021. Note: No estimates are available for Western Sahara as it was not a modeled location in the Global Burden of Disease Study (GBD) 2021.

In Africa, the ASPR, ASDR and ASMR for schistosomiasis in 2021 were 9,461.76 per 100,000 (95% UI 6,900.58 to 12,501.22), 126.43 per 100,000 (80.66 to 211.13) and 1.44 per 100,000 (1.27 to 1.63), respectively. The total number of cases, DALYs and mortality in 2021 were 127 million (91–168 million), 1.5 million (0.92 to 2.58 million) and 11,222 (9,749.48 to 12,756.83), accounting for 84.25%, 87.92% and 87.28% of the global totals for cases DALYs and mortality, respectively (Figure 1 and Table S1).

From 1990 to 2021, schistosomiasis ASPR, ASDR and ASMR in Africa showed an overall downward trend. AAPC analysis indicated that from 1990 to 2021, the ASPR declined at an accelerating rate (Table S2), ASDR consistently decreased (with the fastest decline observed between 2016 and 2019 (−6.07% (95% CI −6.37 to −5.76%))), and the decline rate slowed between 2019 and 2021 (−4.95% (−5.27 to −4.63%)) (Table S3). The ASMR decline decelerated until 2014, after which it significantly accelerated, reaching the maximum rate of decline (−5.50% (−5.60 to −5.40%)) (Table S4).

### 3.2. Regional Level

In the five regions of Africa according to the GBD 2021 study, West Africa had the highest schistosomiasis ASPR and ASDR in 2021, at 14,308.56 per 100,000 (95% UI 10,299.83 to 18,892.56) and 177.69 per 100,000 (108.35 to 303.96), respectively, while North Africa had the lowest rates, with 1,509.2 per 100,000 (1,096.12 to 2,119.55) and 21.51 per 100,000 (13.88 to 36.08). Central Africa had the highest age-standardized mortality rate in 2021 at 3.02 per 100,000 (2.48 to 3.66), while North Africa had the lowest at 0.25 per 100,000 (0.2 to 0.31) (Table S1).

**Figure 2:**
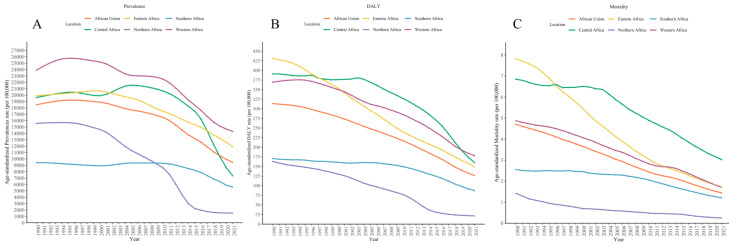
The age-standardized prevalences rate (**A**), age-standardized DALYs rate (**B**), and age-standardized mortality rate (**C**) of schistosomiasis for five African regions from 1990 to 2021. The mortality data begin from 1980.

As shown in Figure 2A, there was an overall declining trend across the five regions of Africa. However, apart from North Africa, the ASPR fluctuated and rose in certain years across the other regions. The ASDR displayed a consistent decline in all regions (Figure 2B), while ASMR decreased in most regions but showed a slower decline after 2012, except for in Central Africa (Figure 2C).

### 3.3. Country Level

**Figure 3:**
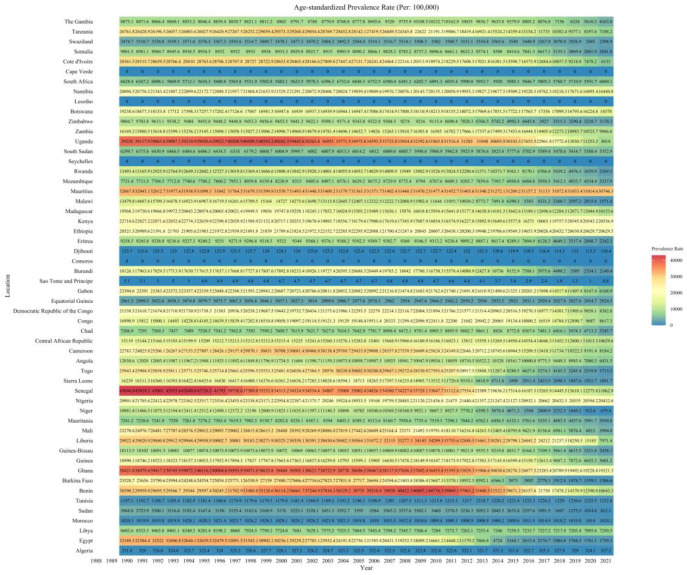
The age-standardized prevalence rates of schistosomiasis for 54 African countries from 1990 to 2021.

Among 54 African countries in 2021, Mauritius had the highest ASPR at 30,746.27 per 100,000 (95% UI 21,636.25 to 41,213.11), while Djibouti had the lowest at 110.39 per 100,000 (77.52 to 1.01) (Fig 3). The Central African Republic had the highest ASDR at 401.6 per 100,000 (289.82 to 540.61), whereas São Tomé and Príncipe had the lowest at 1.51 per 100,000 (0.71 to 2.62) (Figure S1). In terms of mortality, the Central African Republic had the highest ASMR at 9.91 per 100,000 (6.98 to 13.26), while São Tomé and Príncipe had the lowest at 0.05 per 100,000 (0.02 to 0.09) (Figure S2).

As depicted in Figure 2, most countries showed a downward trend in prevalence. However, Benin, Liberia, Burkina Faso, and Cameroon experienced an initial increase followed by a decrease in prevalence. Prevalence rates remained relatively high in Ghana, Uganda, and Senegal (Figure 3). The health burden caused by schistosomiasis was notably severe in Senegal, Ethiopia, the Central African Republic and Uganda (Figure S1). Mortality rates were especially concerning in Ethiopia and the Central African Republic compared to other countries (Figure S2).

### 3.4. Age and Sex Patterns

**Figure 4:**
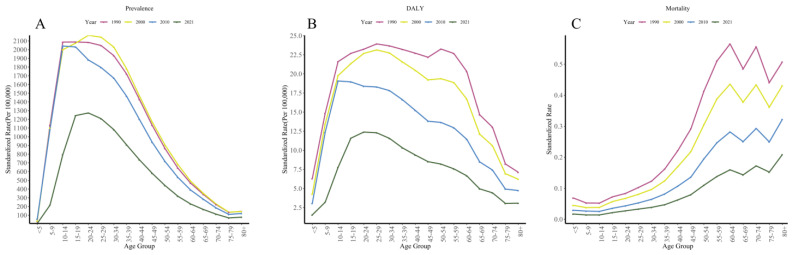
The age-standardized prevalences rate (**A**), age-standardized DALYs rate (**B**), and age-standardized mortality rate (**C**) of schistosomiasis in different age groups in Africa in 1990, 2000, 2010, and 2021. We calculated age-specific standardized rates (Per 100,000) using GBD-provided standard population data.

As shown in Fig. 4, in Africa, for the years 1990, 2000, 2010 and 2021, the ASPR and ASDR both displayed a pattern of first increasing and then decreasing with age, while the ASMR showed a trend of continuous increase followed by fluctuations. Regarding prevalence, the rate gradually increased with age, peaking at 15–34 years (Figure 4A). The distribution of ASDR varied across different age groups over the years, with the highest mortality occurring among the elderly (Figure 4B).

The AAPC results indicate that ASPR, ASDR and ASMR for schistosomiasis decreased across all age groups. The decline in ASPR and ASDR was the fastest in younger age groups, with a moderate decline in middle age groups and the slowest decline in older age groups. The prevalence rate decreased most rapidly in the under-5 age group (−5.48% (95% CI −6.10 to −4.85%)), while the 20–24 age group experienced the slowest decline (−1.58% (−1.70 to −1.45%)) (Table S5). Similarly, the ASDR decline was slowest in the 20–24 age group (-2.00% (−2.21 to −1.91%)) and fastest in the 5–9 age group (−4.81% (−5.06 to −4.56%)). The AAPC results for DALYs rates showed faster declines in both younger and older age groups, with slower declines in middle-aged groups (Table S6). Mortality rates declined relatively quickly in the under-5 age group (-4.05% (−4.01 to −3.94%)) and more slowly in the 80+ age group (−2.61% (−2.73 to −2.50%)), with similar rates of decline across other age groups (Table S7).

**Figure 5:**
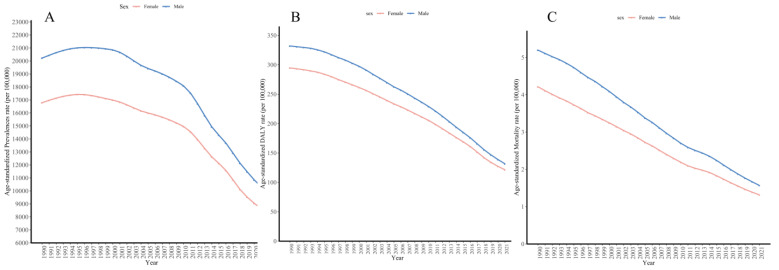
The age-standardized prevalences rate (**A**), age-standardized DALYs rate (**B**), and age-standardized mortality rate (**C**) of schistosomiasis in males and females in Africa from 1990 to 2021. The mortality data began in 1980.

From 1990 to 2021, the age-standardized prevalence rates, DALYs rates and mortality rates for males consistently remained higher than those for females, with both genders showing a downward trend (Figure 5). AAPC analysis revealed that the ASPR for males decreased at a slightly faster rate of -2.17% (95% CI −2.24 to −2.10%) compared to −2.13% (−2.26 to −1.97%) for females (Table S2).

From 1990 to 2021, the ASDR for both males and females in Africa exhibited a continuous decline with a similar overall trend. The decline accelerated gradually from 1990 to 2019, but slowed considerably from 2019 to 2021. The overall AAPC results showed that the decline rate for males (−2.94% (95% CI −2.94 to −2.89%) was slightly faster than for females (−2.82%, (−2.96 to −2.78%)). However, in several periods, such as 1990-2000 and 2016-2019, the decline for females was faster than that for males (Table S3). The ASMR for both males and females in Africa showed a downward trend from 1980 to 2021. Before 2006, the decline of ASMR in females was faster than that in males. After 2006, the decline in male mortality rates outpaced that of females, continuing through to 2021 (Table S4).

### 3.5. Spatial Autocorrelation Analysis

As shown in Figure 1, the ASPR, ASDR and ASMR across African countries in 2021 varied significantly, represented by the gradient of color intensity, indicating a substantial disparity in disease burden among countries.

Global spatial autocorrelation analysis revealed that the ASPR exhibited significant global autocorrelation in 2000 and 2010, while the ASDR and ASMR showed global autocorrelation in 1990, 2000 and 2010. Among the five African regions, the ASMR in North Africa demonstrated global autocorrelation across all four years analyzed.

**Figure 6:**
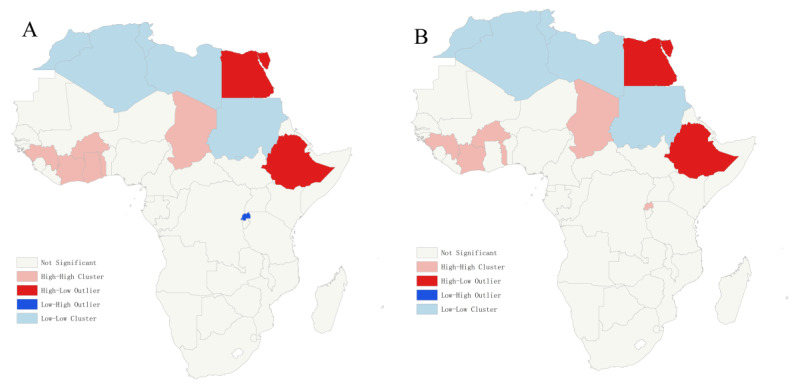
Local autocorrelation analysis of age-standardized prevalence rates of schistosomiasis in Africa for the years 2000 (**A**) and 2010 (**B**).

Due to the lack of data for four countries, including Cape Verde and Lesotho, they were excluded from the analysis. Further spatial autocorrelation analyses of the regions with global autocorrelation show that high-value aggregation of ASPR, ASDR and ASMR mostly occurs in the central and western regions of Africa, such as Côte d’Ivoire and Congo, and the low-value aggregation of ASPR and ASMR occurs in some countries in North Africa over multiple years, such as Algeria (Figure 6, Figure S3, Figure S4, Table S8, Table S9, Table S10), and Ethiopia in 2000 and 2010 (Figure 6). With the exception of North Africa, South Sudan has been identified as a low-value outlier for ASMR over many years (Figure S4). 

### 3.6. Model Prediction

The results of the time series models indicate that the optimal model varies for different indicators among the five algorithms, which include ARIMA, PROPHET, MARS, BSTS, and DLM. The best model is here selected based on six evaluation metrics—MSE, RMSE, MAPE, MARE, SMAPE and R². The goal is to choose a model with low error and high R².

**Figure 7:**
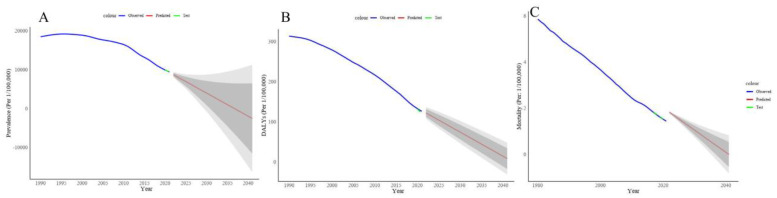
Forecast of the age-standardized prevalences rate (**A**), age-standardized DALYs rate (**B**) and age-standardized mortality rate (**C**) of schistosomiasis in Africa, 2022–2041. In the figure, blue represents actual values, green represents predicted values for the test set from 2020–2021, red represents predictions for the disease burden of schistosomiasis from 2022–2041, the dark gray area represents the 95% confidence interval, and the gray area represents the 80% confidence interval. The mortality data begin in 1980.

The prediction results are shown in Figure 7. Overall, the ARIMA model performs the best for predicting schistosomiasis ASPR in Africa. The auto.arima function indicates that the model with the smallest Akaike Information Criterion (AIC) and Bayesian Information Criterion (BIC) for prevalence prediction is ARIMA(3, 2, 0) (AIC = 312.77, BIC = 318.09). The Ljung–Box test for the residuals shows a first-order result (P = 0.76), indicating no statistical significance and suggesting white noise. In the Figure 7A, blue represents actual values, green represents predicted values for the test set from 2020–2021, red represents predictions for the ASPR of schistosomiasis from 2022–2041, the dark gray area represents the 95% confidence interval, and the gray area represents the 80% confidence interval.

The forecast indicates that by 2030, the age-standardized prevalence rate for schistosomiasis in Africa will be 3.99%, significantly exceeding the WHO target of reducing global prevalence to below 1% by 2030 for elimination. Based on the predicted trend, schistosomiasis prevalence in Africa will reach approximately 0.43% by 2036, achieving the WHO’s elimination standard (Figure 7A).

Among the five models used for predicting ASDR, the ARIMA (0, 1, 0) model (AIC = 169.96, BIC = 172.69) performed best. The Ljung–Box test for the residuals yielded a lag of 6 with P = 0.09, indicating no statistical significance and suggesting white noise. In Figure 7B, blue represents actual values, green represents predicted values for the test set from 2020–2021, red represents predictions for schistosomiasis ASDR from 2022–2041, and the confidence interval areas are represented as previously mentioned. The predictions show a continuous downward trend in DALYs due to schistosomiasis over the next 20 years. By 2030 and 2041, the DALYs due to schistosomiasis in Africa will be 74.44/100,000 and 8.67/100,000, respectively. According to the prediction model, the disease burden caused by schistosomiasis will continue to decrease in the future (Figure 7B). The DLM model is identified as the best predictor for the ASMR of schistosomiasis in Africa, showing that by 2041, deaths caused by schistosomiasis will be reduced to zero (Figure 7C).

## 4. Discussion

Our study found that in 2021, the overall burden of schistosomiasis in Africa was high, accounting for the majority of global cases. Specifically, the prevalence, DALY and death toll from schistosomiasis in Africa accounted for 84.25%, 87.29%, and 87.28% of global totals, respectively, which aligns with previous research findings [11]. Among the regions, West Africa exhibited the highest the ASPR and ASDR, while North Africa had the lowest. In contrast, North Africa’s schistosomiasis prevalence was reduced in 1960 through irrigation changes due to the construction of the Aswan Dam [19].

Based on the GBD 2021 study, we analyzed the disease burden of schistosomiasis in Africa and its different regions for 2021, as well as the trends from 1990 to 2021. Furthermore, we constructed five different models to select the optimal one for predicting future disease burden in Africa. Additionally, we employed spatial autocorrelation analysis to reveal the geographic clustering characteristics of schistosomiasis prevalences in different African regions. From 1990 to 2021, the burden of schistosomiasis in Africa showed a declining trend. Over the past decade, with the WHO’s assistance in implementing mass drug administration using praziquantel, significant progress has been made in schistosomiasis control [20]. However, there remains a considerable gap between the current situation and the WHO’s goal to eliminate schistosomiasis as a public health issue by 2030, defined as a prevalence of severe schistosomiasis infection below 1% [21], due to high infection rates, low coverage of mass drug administration, and recurrent exposure to contaminated water, combined with climate and socioeconomic factors, as a result of which residents often experience repeated infections [6,22]. Therefore, besides mass drug administration, it is crucial for many countries to prioritize strategies such as implementing snail control, improving sanitation conditions, and ensuring access to safe, clean water [23].

Our study results show that the peak prevalence of schistosomiasis in 2021 was concentrated in the 15–29 age group. The ASPR values for all age groups were higher for males than for females, which may be related to differences in water exposure due to cultural, religious, occupational, or social roles, men typically having more frequent contact with water [24,25]. However, under conditions of gender inequality and entrenched cultural constraints, women often seek treatment later than men, or do not seek it at all [26,27,28], resulting in a slightly faster decline of the ASPR in males compared to females. Although the ASPR of males is higher than that of females, the ASDR for females peak slightly higher than that for males, with a slower decline in DALYs among women of reproductive age. This is associated with risks such as increased HIV infection [29,30] and gynecological complications like infertility and ectopic pregnancy, due to schistosomiasis affecting the reproductive system. It is estimated that up to 56 million women in sub-Saharan Africa are affected by female genital schistosomiasis, with nearly 20 million new cases projected over the next decade if left untreated [31]. With the increasing attention being paid to female genital schistosomiasis, the United Nations Programme on HIV/AIDS published a report in 2019 titled "Don’t Ignore: Female Genital Schistosomiasis and HIV" [32], emphasizing the need for targeted measures for women, including community health education, increased access to preventive chemotherapy, HPV vaccination, and screening for female genital schistosomiasis [33].

The AAPC results indicate that the prevalence rate, DALYs rate, and mortality rate of schistosomiasis are decreasing rapidly among younger age groups (0–9 years). Preventive chemotherapy is primarily focused on school-aged children, who are considered to be at high risk of infection. Since Merck & Co., Inc. committed to donating 250 million doses of praziquantel annually in 2005, more children have received treatment. The WHO’s latest data show that 43.3% of school-aged children needing preventive chemotherapy are being treated, while only 29.9% of all populations requiring it are receiving treatment [9]. In addition to children, populations at risk of exposure to contaminated water, such as farmers and women washing clothes in rivers, should also have more treatment opportunities. 

The global autocorrelation results for schistosomiasis in Africa indicate the significant global autocorrelation of ASPR in 2000 and 2010, while the ASDR and ASMR showed global autocorrelation in 1990, 2000 and 2010. Further local autocorrelation analyses reveal high clustering primarily in parts of Central and West Africa, with low clustering concentrated in North Africa. Ethiopia consistently shows high prevalence across different years, and several studies have shown that the prevalences of schistosomiasis is related to economy, temperature, precipitation and vegetation index, increased precipitation, suitable temperatures, and rainwater inundation, all of which may explain the lower prevalence and disease burden in North Africa [29,34,35]. In addition, the prevalence of schistosomiasis in many countries in North Africa, such as Burkina Faso and Algeria, has been declining rapidly with the implementation of large-scale praziquantel donation and schistosomiasis surveillance and control programs [36,37]. There was no global autocorrelation for schistosomiasis in Africa by 2021. The global autocorrelation analysis for Africa’s five regions showed that North Africa exhibited global autocorrelation in mortality rates across four different years, while no correlation was found in other African regions.

When comparing the predictive models, ARIMA emerged as the best model for prevalence prediction, indicating that schistosomiasis prevalence in Africa will continue to decline, though it is projected to reach an age-standardized prevalence of 3.99% by 2030, well above the WHO’s elimination target. In the future, Africa may need to expand treatment coverage beyond just school-aged children to include other high-risk groups (e.g., fishermen, farmers) and increase the frequency of preventive chemotherapy from once to twice a year [38]. The ongoing transmission of schistosomiasis in sub-Saharan Africa is driven by various factors, including poverty limiting access to safe water and adequate sanitation facilities, geographic expansion due to climate change, ecological changes relating to human-made irrigation systems, reservoirs and dams, and the fact that nearly 76% of Africa’s population lives near open water bodies [6,9,39]. Currently, most African countries focus their schistosomiasis control efforts on community-based preventive chemotherapy, with an emphasis on mass drug administration using praziquantel, a broad-spectrum anthelmintic, to reduce morbidity, particularly in school-age children who are at high risk [39,40]. However, challenges such as poor treatment adherence, schistosomiasis reinfection, inadequate chemotherapy coverage, praziquantel resistance and climate changes that favor the growth of snail intermediate hosts have contributed to the ongoing severity of schistosomiasis in Africa [39,41,42]. Furthermore, enhancing access to clean water, improving environmental sanitation and promoting personal hygiene, mapping contaminated water sources for precise snail control and expanding community engagement in information, education and communication activities are crucial multi-strategy approaches to raising health awareness and reducing infection rates, which are all essential for accelerating schistosomiasis elimination efforts [43]. 

This study has certain limitations. First, the GBD 2021 study relies on mathematical and epidemiological models for data estimation, which may introduce biases. Second, the GBD study contains schistosomiasis data from all over the world, but does not classify schistosomiasis in detail, and all schistosomiasis DALY values are calculated using the same disability weight value, which limits the accurate assessment of the disease burden of different types of schistosomiasis and cannot accurately reflect the types and prevalences of schistosomiasis in different countries. Future studies may consider classifying different types of schistosomiasis and calculating disability weight in order to more accurately assess the burden of disease due to different types of schistosomiasis. Lastly, this study’s model does not account for the impacts of temperature, precipitation and other factors on changes in disease burden.

## 5. Conclusions

Our study found that the disease burden caused by schistosomiasis in Africa decreased from 1990 to 2021, with significant declines in related mortality and DALYs rates. The rates of prevalence, mortality and DALYs related to schistosomiasis have decreased more rapidly among males and younger age groups. However, model predictions indicate that by 2030, the ASPR of schistosomiasis will be 3.99%, suggesting that further efforts are needed to meet the WHO’s 2030 elimination target. This is particularly important in high-burden regions and populations, such as West Africa. Finally, policy-makers should consider changing current strategies and investing more in eliminating schistosomiasis. For example, increasing the coverage and frequency of preventive chemotherapy, implementing targeted snail control, and integrating health education programs are crucial for accelerating the elimination of schistosomiasis in Africa.

## Data Availability

Data are available in a public, open-access repository. The data used for the analyses are publicly available on the Institute of Health Metrics and Evaluation website (VizHub—GBD Results).

## Supplementary Materials

The following supporting information can be downloaded at: https://www.mdpi.com/article/10.3390/tropicalmed10020042/s1.
