# Supplementary material for: Schistosomiasis Burden and Trend Analysis in Africa: Insights from the Global Burden of Disease Study 2021
Source: Trop Med Infect Dis. 2025 Feb 3;10(2):42. doi: 10.3390/tropicalmed10020042 (PMC11860299; doi:10.3390/tropicalmed10020042)
Supplement: Supplementary file 1 [file tropicalmed-10-00042-s001.zip › tropicalmed-3409915-supplementary/Supporting information of figure.pdf]

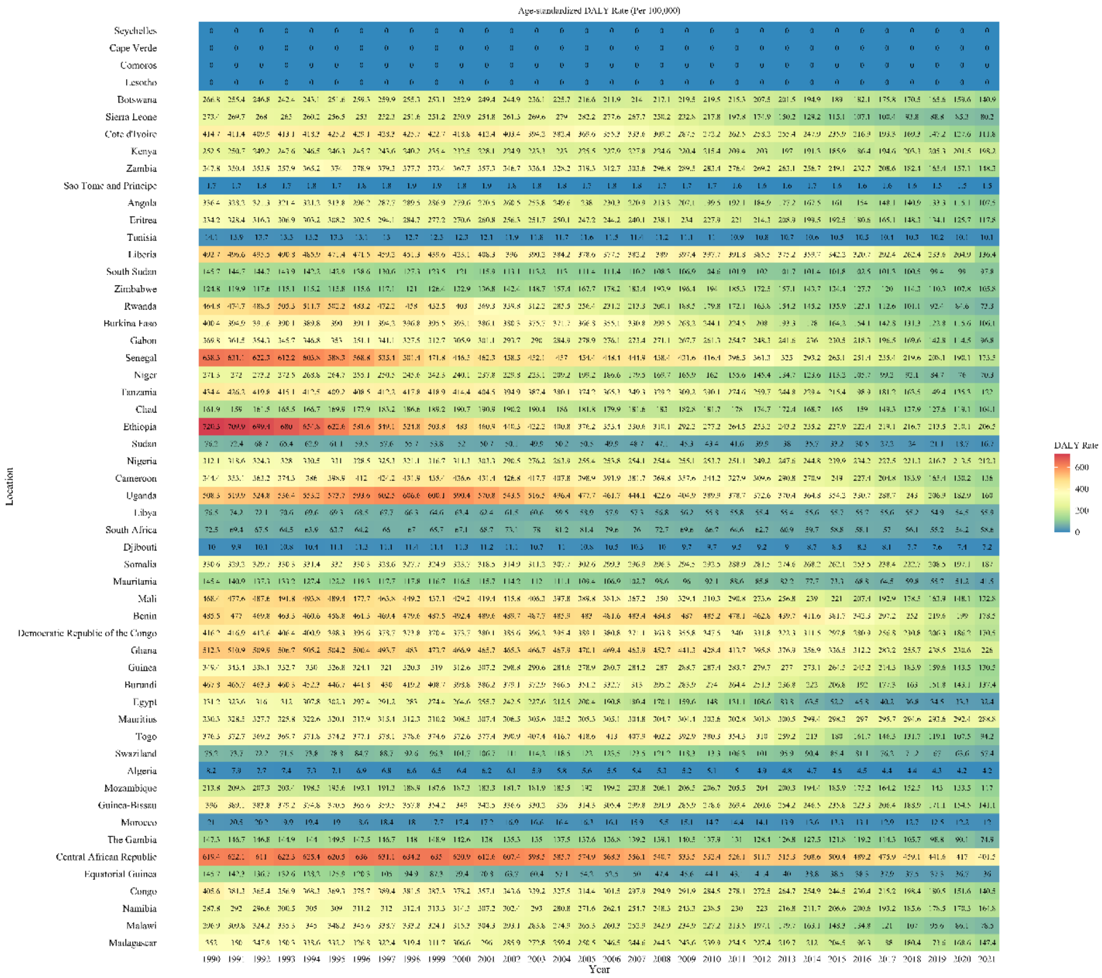

Figure S1. The age-standardized DALYs rate of schistosomiasis for 54 African countries from 1990 to 2021

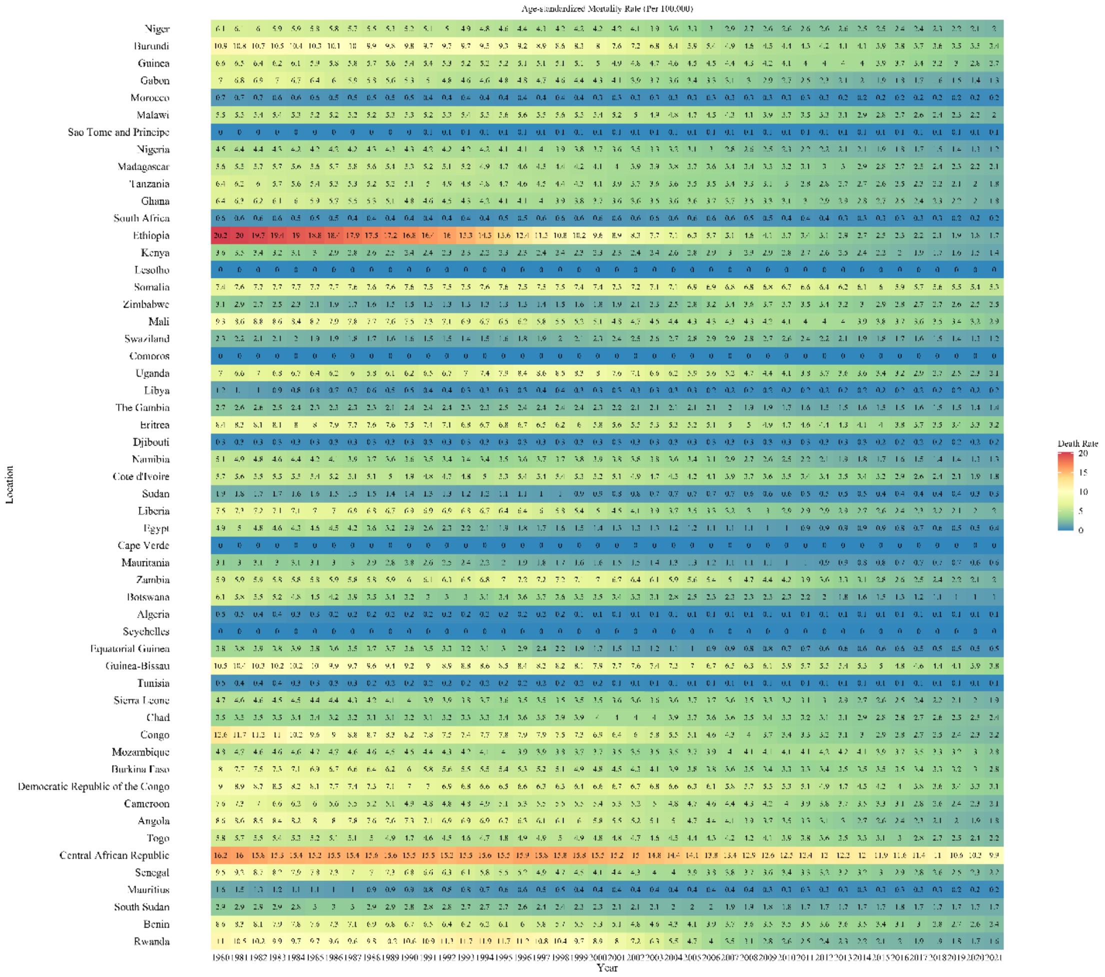

Figure S2. The age-standardized mortality rate of schistosomiasis for 54 African countries from 1990 to 2021

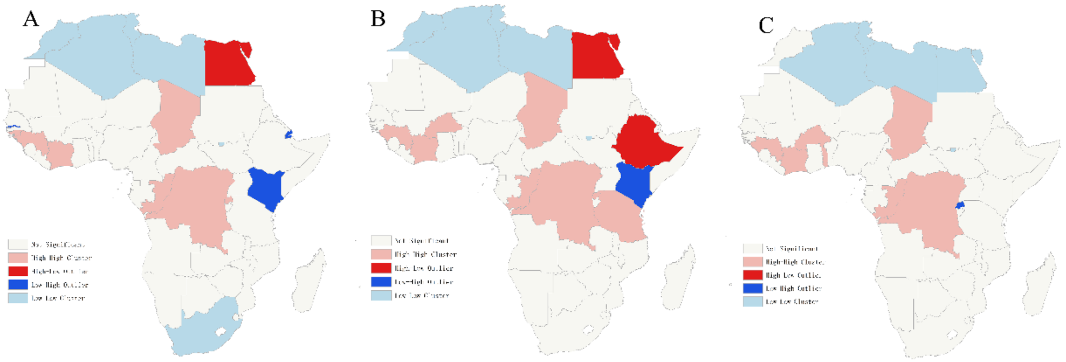

Figure S3. Local autocorrelation analysis of ASDR in Africa for the years 1990, 2000, 2010

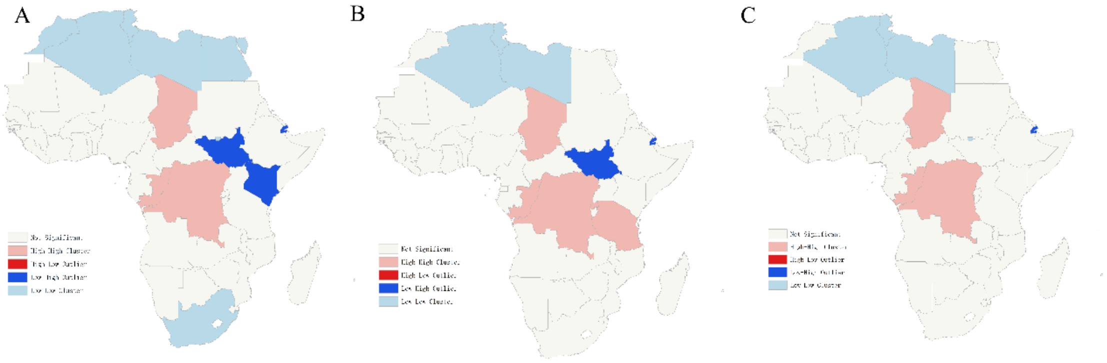

Figure S4. Local autocorrelation analysis of ASMR in Africa for the years 1990, 2000, 2010
