# Supplementary material for: Schistosomiasis Burden and Trend Analysis in Africa: Insights from the Global Burden of Disease Study 2021
Source: Trop Med Infect Dis. 2025 Feb 3;10(2):42. doi: 10.3390/tropicalmed10020042 (PMC11860299; doi:10.3390/tropicalmed10020042)
Supplement: Supplementary file 1 [file tropicalmed-10-00042-s001.zip › tropicalmed-3409915-supplementary/Supporting information of Table.pdf]

Table S1. Estimated number and ASR of prevalence, DALYs, and mortality of schistosomiasis in 1990 and 2021

Region	1990						2021					
	Prevalence		DALYs		Mortality		Prevalence		DALYs		Mortality	
	Number	ASR	Number	ASR	Number	ASR	Number	ASR	Number	ASR	Number	ASR
African Union	110732175.98 (91103993.17, 131173338.26)	18495.51 (15166.27 , 22183.82)	1629752.98 (1146954.98, 2530831.07)	313.6 (230.85 , 461.15)	16355.52 (14378.19 , 18447.94)	4.71 (4.18 , 5.25)	127535371.8 (91356330.47 , 167805780.44)	9461.76 (6900.58, 12501.22)	1535379.9 (921966.79 , 2581477.28)	126.43 (80.66, 211.13)	11222 (9749.48 , 12756.83)	1.44 (1.27 , 1.63)
Central Africa	12164437.71 (9574316.24, 14776443.59)	19626.48 (15444.41 , 23812.17)	200040.75 (144351.04, 304302.97)	390.68 (294.74 , 561.01)	2341.43 (1909.32, 2807.82)	6.85 (5.65 , 8.23)	11631815.07 (8035099.15, 15843335.75)	7379.21 (5189.5, 10289.32)	199853.9 (141311.08 , 304172.66)	159.78 (117.77 , 228.26)	2441.46 (1948.45 , 2966.35)	3.02 (2.48 , 3.66)
Eastern Africa	32750686.07 (26701152.95, 39339599.73)	19908.61 (16229.06 , 23797.34)	574566.89 (425826.29, 842673.46)	431.42 (332.76 , 592.9)	6952.79 (5964.78, 8221.09)	7.83 (6.73 , 9.21)	44203214.35 (32146225.86 , 57148200.76)	11923.37 (8834.03, 15419.27)	488726.63 (287736.2, 862148.57)	149.21 (94.85, 253.64)	3346.91 (2977.71 , 3809.85)	1.72 (1.53 , 1.95)
Northern Africa	18620783.31 (17783563.9, 19503507.92)	15572.7 (14885.84 , 16317.27)	179285.64 (107282.81, 310497.32)	162.85 (103.67 , 271.18)	930.22 (788.39, 1096.81)	1.42 (1.19 , 1.69)	3194927.38 (2297968.91, 4539482.89)	1509.2 (1096.12, 2119.55)	42941.89 (27019.35, 74086.65)	21.51 (13.88, 36.08)	375.14 (298.2, 467.66)	0.25 (0.2, 0.31)
Southern Africa	8547928.29 (6391606.88, 10953658.88)	9420.42 (7126.23, 12225.52)	135982 (94747.63, 208216)	170.73 (126.34 , 250.61)	1428.48 (1213.64, 1650.05)	2.56 (2.2, 2.97)	10095872.02 (7198299.59, 13817353.73)	5603.26 (4057.96, 7690.46)	141764.01 (93413.29, 227692.82)	87.55 (60.11, 137.13)	1402.07 (1145.21 , 1676.51)	1.21 (1.01 , 1.43)
West	38648340.61	23934.72	539877.7	369.56	4702.6	4.88	58409542.98	14308.56	662093.47	177.69	3656.41	1.71

ern Africa	(30097445.61, 47829870.59)	(18609.52 , 30047.45)	(361108.1, 849702.15)	(258.41 , 565.35)	(4045.89, 5398.55)	(4.28 , 5.61)	(41940524.51 , 76927837.38)	(10299.83 , 18892.56)	(378540.41 , 1140463.76)	(108.35 , 303.96)	(3026.11 , 4300.42)	(1.46 , 1.98)

Table S2. AAPC of the age-standardized prevalence rate of schistosomiasis in Africa by gender from 1990 to 2021

Sex		Year	Change95%CI(%)	"Test Statistic"	"P-Value"
			Age Standardized Prevalence Rate		
Both	APC	1990–1995	0.79(0.61–0.97)	9.35	0.00
		1995–2000	−0.38(−0.62 ~ −0.13)	−3.23	0.01
		2000–2010	−1.28(−1.35 ~ −1.20)	−34.23	0.00
		2010–2016	−4.55(−4.77 ~ −4.33)	−43.11	0.00
		2016–2021	−5.71(−5.96 ~ −5.46)	−46.24	0.0
	AAPC	1990–2021	−2.18(−2.25 ~ −2.10)	−55.92	0.00
Female	APC	1990–1995	0.79(0.64−0.94)	11.66	0.0
		1995–2000	−0.58(−0.79 ~ −0.37)	−6.03	0.00

		2000–2008	−1.17(−1.26 ~ −1.08)	−27.20	0.0
		2008–2011	−1.94(−2.72 ~ −1.15)	−5.32	0.00
		2011–2016	-4.57(-4.82 ~ -4.31)	-38.15	0.00
		2016–2019	−6.29(−7.18 ~ −5.40)	−14.91	0.00
		2019–2021	−4.67(−5.64 ~ −3.69）	−10.16	0.00
	AAPC	1990–2021	−2.13(−2.26 ~ −1.97)	−30.91	0.00
Male	APC	1990–1995	0.79(0.62 ~ 0.96)	9.74	0.00
		1995–2000	−0.21(−0.44 ~ 0.03)	−1.83	0.08
		2000–2010	−1.35(−1.43 ~ −1.28)	−37.88	0.00
		2010–2016	−4.70(−4.91 ~ −4.49)	−45.67	0.00
		2016–2021	−5.53(−5.78 ~ −5.29)	−45.84	0.00
	AAPC	1990–2021	−2.17(−2.24 ~ −2.10)	−57.64	0.00

Table S3. AAPC of the age-standardized DALYs rate of schistosomiasis in Africa by gender from 1990 to 2021

Sex	Model	Year	Change95%CI(%)	Test Statistic	P-Value
			Age-standardized DALYs rate		
Both	APC	1990–1994	−0.50 (−0.58 ~ −0.42)	−14.07	0.00
		1994–2000	−1.54 (−1.60 ~ −1.48)	−57.35	0.00
		2000–2007	−2. (−2.414 ~ −2.321)	−109.14	0.00
		2007–2011	−2.89 (−3.03 ~ −2.74)	−41.39	0.00
		2011–2016	−4.34 (−4.43 ~ −4.24)	−96.45	0.00
		2016–2019	−6.07 (−6.37 ~ −5.76)	−41.87	0.00
		2019–2021	−4.95(−5.27 ~ −4.63)	−33.16	0.00
	AAPC	1990–2021	−2.89(−2.93 ~ −2.85)	−128.79	0.00
Female	APC	1990–1994	−0.58 (−0.66 ~ −0.51)	−16.86	0.00
		1994–2000	−1.57 (−1.62 ~ −1.51)	−59.86	0.00
		2000–2007	−2.26 (−2.30 ~ −2.21)	−106.37	0.00
		2007–2011	−2.80 (−2.94 ~ −2.65)	−41.43	0.00
		2011–2016	−4.13 (−4.22 ~ −4.04)	−94.89	0.00
		2016–2019	−6.12 (−6.41 ~ −5.83)	−44.30	0.00
		2019–2021	−4.67 (−4.97 ~ −4.37)	−33.43	0.00
	AAPC	1990–2019	−2.822(−2.86 ~ −2.78)	−131.40	0.00

Male	APC	1990–1994	−0.430 (−0.52 ~ −0.34)	−10.22	0.00
		1994–2000	−1.508 (−1.58 ~ −1.44)	−47.89	0.00
		2000–2007	−2.459 (−2.51 ~ −2.41)	−96.85	0.00
		2007–2011	−2.953 (−3.13 ~ −2.78)	−36.16	0.00
		2011–2016	−4.517(−4.63 ~ −4.41)	−86.34	0.00
		2016–2019	−5.998(−6.35 ~ −5.64)	−35.62	0.00
		2019–2021	−5.209(−5.58 ~ −4.84)	−30.02	0.00
	AAPC	1990–2019	−2.944(−2.94 ~ −2.89)	−112.57	0.00

Table S4. AAPC of the age-standardized mortality rate of schistosomiasis in Africa by gender from 1980 to 2021

Sex	Model	Year	Change95%CI(%)	Test Statistic	P-Value
		Age-standardized mortality rate			
Both	APC	1980–1995	−2.16(−2.19 ~ −2.12)	−129.71	0.00
		1995–2001	−2.86(−3.01 ~ −2.70)	−36.92	0.00
		2001–2006	−3.74(−3.95 ~ −3.52)	−35.21	0.00
		2006–2011	−4.50(−4.71 ~ −4.30)	−44.54	0.00

		2011–2014	−2.82(−3.49 ~ −2.15)	−8.58	0.00
		2014–2021	−5.50(−5.60 ~ −5.40)	−110.19	0.00
	AAPC	1980–2021	−3.37(−3.43 ~ −3.30)	−99.84	0.00
Female	APC	1980–1985	−1.882(−2.08 ~ −1.68)	−19.56	0.00
		1985–1999	−2.550(−2.59 ~ −2.51)	−123.41	0.00
		1999–2003	−3.143(−3.51 ~ −2.78)	−17.45	0.00
		2003–2006	−3.78(−4.47 ~ −3.08)	−11.09	0.00
		2006–2011	−4.44(−4.66 ~ −4.22)	−40.88	0.00
		2011–2014	−2.74(−3.45 ~ −2.01)	−7.75	0.00
		2014–2021	−5.28(−5.38 ~ −5.18)	−104.28	0.00
	AAPC	1980–2021	−3.33(−3.42 ~ −3.25)	−73.89	0.00
Male	APC	1980–1988	−2.22(−2.31 ~ −2.14)	−55.42	0.00
		1988–1994	−1.67(−1.83 ~ −1.51)	−21.28	0.00

	1994–2000	−2.78(−2.93 ~ −2.63)	−37.86	0.00
	2000–2006	−3.77(−3.91 ~ −3.64)	−56.56	0.00
	2006–2011	−4.53(−4.70 ~ −4.35)	−52.97	0.00
	2011–2014	−2.87(−3.44 ~ −2.29)	−10.23	0.00
	2014–2021	−5.67(−5.75 ~ −5.60)	−143.85	0.00
AAPC	1980–2021	−3.38(−3.44 ~ −3.32)	−108.17	0.00

Table S5. AAPC of the age-standardized prevalence rate of schistosomiasis in Africa by age from 1990 to 2021

"AgeGroup"	"Joinpoint Model"	"AAPC"	"TestStatistic"	"P-Value"
Age-standardized prevalence rate				
<5	4	−5.47 (−6.10 ~ −4.85)	−16.72	0.00
5 ~ 9	5	−5.23 (−5.55 ~ −4.90)	−30.42	0.00
10 ~ 14	5	−3.10 (−3.24 ~ −2.95)	−41.54	0.00
15 ~ 19	4	−1.66 (−1.75 ~ −1.56)	−33.85	0.00
20 ~ 24	5	−1.58 (−1.70 ~ −1.45)	−24.72	0.00
25 ~ 29	5	−1.68 (−1.81 ~ −1.56)	−26.16	0.00
30 ~ 34	5	−1.87 (−2.01 ~ −1.73)	−25.56	0.00
35 ~ 39	5	−2.06 (−2.19 ~ −1.92)	−30.10	0.00

40 ~ 44	3	−2.14 （−2.20 ~ −2.07）	−62.32	0.00
45 ~ 49	3	−2.17 （−2.26 ~ −2.07）	−45.21	0.00
50 ~ 54	6	−2.16 （−2.32 ~ −2.00）	−25.84	0.00
55 ~ 59	6	−2.24 （−2.43 ~ −2.06）	−23.29	0.00
60 ~ 64	6	−2.30 （−2.53 ~ −2.07）	−19.57	0.00
65 ~ 69	5	−2.29 （−2.43 ~ −2.16）	−33.10	0.00
70 ~ 74	4	−2.21 （−2.33 ~ −2.10）	−36.85	0.00
75 ~ 79	6	−2.12(−2.20 ~ −2.03)	−48.04	0.00
80+	6	−1.97(−2.03 ~ −1.90)	−58.41	0.00

Table S6. AAPC of the age-standardized DALYs rate of schistosomiasis in Africa by age from 1990 to 2021

"AgeGroup"	"Joinpoint Model"	"AAPC"	"Test Statistic"	"P-Value"
Age-standardized DALYs rate				
<5	6	−4.43 （−4.61 ~ −4.25）	−46.70	0.00
5 ~ 9	6	−4.81(−5.060 ~ −4.56)	−37.05	0.00
10 ~ 14	6	−3.28 （−3.468 ~ −3.10）	−34.11	0.00
15 ~ 19	5	−2.15 （−2.21 ~ −2.09）	−68.48	0.00
20 ~ 24	6	−2.01 （−2.10 ~ −1.92）	−41.88	0.00
25 ~ 29	6	−2.13 （−2.20 ~ −2.05）	−53.86	0.00
30 ~ 34	5	−2.29(−2.35 ~ −2.23)	−72.92	0.00
35 ~ 39	6	−2.59(−2.64 ~ −2.54)	−95.66	0.00
40 ~ 44	5	−2.82(−2.90 ~ −2.75)	−72.90	0.00
45 ~ 49	6	−3.05(−3.15 ~ −2.95)	−60.25	0.00

50 ~ 54	6	−3.35(−3.38 ~ −3.26)	−102.47	0.00
55 ~ 59	3	−3.54(−3.57 ~ −3.44)	−108.57	0.00
60 ~ 64	6	−3.54(−3.61 ~ −3.46)	−89.923	0.00
65 ~ 69	6	−3.43(−3.54 ~ −3.36)	−92.37	0.00
70 ~ 74	6	−3.42(−3.52 ~ −3.32)	−65.45	0.00
75 ~ 79	4	−3.17(−3.26 ~ −3.08)	−67.10	0.00
80+	5	−2.67(−2.77 ~ −2.56)	−49.72	0.00

Table S7. AAPC of the age-standardized mortality rate of schistosomiasis in Africa by age from 1980 to 2021

"AgeGroup"	"Joinpoint Model"	"AAPC"(95%CI)	"Test Statistic"	"P-Value"
Age-standardized mortality rate				
<5	4	−4.02(−4.09 ~ −3.94)	−98.76	0.00
5 ~ 9	4	−3.8287(−3.97 ~ −3.69)	−52.06	0.00
10 ~ 14	6	−3.68(−3.82 ~ −3.54)	−51.85	0.00
15 ~ 19	7	−3.44(−3.55 ~ −3.33)	−59.11	0.00
20 ~ 24	7	−3.17(−3.29 ~ −3.05)	−49.15	0.00
25 ~ 29	6	−3.2224(−3.38 ~ −3.06)	−38.90	0.00
30 ~ 34	4	−3.22(−3.35 ~ −3.10)	−49.70	0.00
35 ~ 39	7	−3.46(−3.60 ~ −3.32)	−48.94	0.00
40 ~ 44	6	−3.61(−3.70 ~ −3.51)	−72.54	0.00
45 ~ 49	7	−3.71(−3.82 ~ −3.60)	−65.23	0.00
50 ~ 54	5	−3.78(−3.90 ~ −3.66)	−60.79	0.00
55 ~ 59	6	−3.72(−3.87 ~ −3.58)	−49.51	0.00
60 ~ 64	6	−3.66(−3.73 ~ −3.58)	−95.36	0.00

65 ~ 69	7	−3.47(−3.59 ~ −3.35)	−56.38	0.00
70 ~ 74	7	−3.19(−3.31 ~ −3.07)	−50.90	0.00
75 ~ 79	6	−2.99(−3.12 ~ −2.86)	−44.48	0.00
80+	7	−2.61(−2.73 ~ −2.50)	−43.55	0.00

Table S8. Local autocorrelation analysis of ASPR in Africa for the years 1990, 2000, 2010, and 2021

Year	H-H	L-L	H-L	L-H	No
2010	Cape verde<br>Burkina Faso<br>Togo	Algeria<br>Liyba<br>Tunisia	Ethiopia		
2000	Cape verde<br>Burkina Faso<br>Togo<br>Ghana<br>Guinea	Algeria<br>Liba<br>Tunisia	Ethiopia	Rwanda	
1990	Cape verde<br>Burkina Faso<br>Togo<br>Ghana<br>Guinea	Algeria<br>Liyba<br>Tunisia<br>Morocco		Rwanda<br>Guinea issau<br>Gambia	

Table S9. Local autocorrelation analysis of ASDR in Africa for the years 1990, 2000, 2010, and 2021

Year	H-H	L-L	H-L	L-H	N

					o
2010	Guinea、Ivory Coast、Burkina Faso、Togo、Congo	Morocco、Algeria、Liyba 、 Tunisia 、Egypt			
2000	Ivory Coast 、 Burkina Faso、Togo、Congo、Democratic of Republic Congo,Guinea,Republic of Tanzania	Morocco、Algeria、Liyba 、 Tunisia 、Egypt	Ethiopia	South Sudan	
1990	Ivory Coast 、 Burkina Faso 、 Rwanda 、Democratic Republic of the Congo、Guinea	Morocco、Algeria、Tunisia,Libya,South Africa	Egypt	South Sudan 、Gambia、Djibouti	

Table S10. Local autocorrelation analysis of ASMR in Africa for the years 1990, 2000, 2010, and 2021

Year	H-H	L-L	H-L	L-H	No
2010	Congo 、Democratic of Republic Congo	Algeria 、Liyba 、Tunisia 、Egypt,Morocco		South Sudan	
2000	Congo 、Democratic of	Algeria 、Liyba 、		SouthSudan、Republic of Tanzania	

	Republic Congo	Tunisia、Egypt Morocco			
1990	Congo Democratic of Republic Congo	Algeria 、 Liyba 、 Tunisia、Egypt South Africa,Morocco		South Sudan、Djibouti	
